# Book Reviews

**Published:** 1827-08

**Authors:** 


### CRITICAL ANALYSES.

Quæ laudanda forent, et quæ culpanda, vicissim
Illa, prius, cretâ; mox hæc, carbone, *notamus.*—PERSIUS.

*Principles of Medical Science and Practice.* By HARDWICKE SHUTE, M.D. Physician to the General Infirmary, and to the County and City Lunatic Asylum, Gloucester. Vols. I. and II. —8vo. Longman and Co. London, 1826.

As Dr. SHUTE has undertaken a task of no common magnitude, we cannot be surprised at the size of the volumes with which he has presented us. It would be impossible, even with any powers of condensation, to give a satisfactory abstract of nearly twelve hundred pages within the limits of an ordinary article: the author must, therefore, excuse us if we pass over the volume on Physiology, that we may be enabled to devote more space and attention to that which he has dedicated to pathological considerations. We must be allowed to observe with respect to the physiological part of the work, that it is more calculated for the perusal of the practitioner than the student. It consists principally of comments upon the opinions of different physiologists, interspersed indeed with a few suggestions peculiar to the author. We cannot, however, venture to say that we have detected any originality of sufficient importance to have justified the addition of so large a volume to the works we already possess upon the same subject.

The volume on Pathology commences with a few pages of preliminary observations.

It is observed, that if we inquire into the meaning of the word *disease*,—if we ask ourselves what precise idea is attached to the term,—the definition, though apparently simple, will be found to convey no very precise information. Disease is said to be a deviation from health; but it evidently does not add much to our knowledge to say that one thing is a deviation from another, unless we be able to state what that other is. It is impossible, however, to define, and difficult even to describe, the precise meaning of the word *health*. "In order to approach as nearly as possible to the definition required, we are under the necessity of using terms which have a certain *latitude* of meaning; and the natural consequence is, that each individual adopts the explanation which is most consistent with his own peculiar views of the subject." It is to be remembered that the constitution is frequently placed under extraordinary circumstances, without the health of the system being at all affected. Phenomena, corresponding with this *alteration* in the state of the system will then occur, but are not deviations from health, and do not constitute disease. The pulse of an individual, for example, when at rest, may beat sixty times in a minute; may be increased to eighty by the exercise of walking, or to one hundred by that of running. A pulse of one hundred is a great *deviation* from that of sixty, but is not disease. In order, therefore, to determine what constitutes a diseased action, we must well consider the circumstances under which the constitution is placed at the time.

"Inordinate action is morbid only when disproportioned to its necessity; a pathological principle too often neglected in practice, and uniformly disregarded by those who prescribe for symptoms, and affect to despise all theoretical opinions, which, if the real truth were disclosed, they are too indolent to learn, or too ignorant to understand. There is, in point of fact, no fixed standard of health, nature having provided for a certain range of action, which is not only consistent with the powers of the constitution, but, in many instances, necessary to their continuance and support. Without an intimate acquaintance with the natural extent of these powers,—without a knowledge of the changes and varieties of action which are compatible with a healthy state of the system, and consistent with the peculiar structure of the animal frame,—without a knowledge of the objects and purposes for which these actions are established, it is impossible to ascertain the boundary which forms the line of demarcation between health and disease; and thus physiology, or an acquaintance with the healthy actions of the system, becomes the foundation of all practical medicine." (P. 4.)

The author might also have added, that not only does the rapidity of the circulation deviate from what is considered the healthy standard in the same individual, under different circumstances, without the existence of disease, but it occasionally happens that there is the same departure from the common standard of the circulation in particular individuals at all times. We are acquainted with two men who present all the appearances of health, who are capable of enduring bodily fatigue and mental excitement in perhaps even more than an ordinary degree. In one the pulse is rarely more than fifty-five, while in the other it beats upwards of one hundred strokes in a minute. In either of these cases, if the pulse were found at seventy, it would be considered either an indication of disease, or of some momentary excitement in the one case, or depression in the other. It is desirable, therefore, that the practitioner should be on his guard against these occasional idiosyncrasies of constitution, which might, without some precaution, lead him into error.

Dr. Shute is not inclined to undervalue anatomical knowledge, but he goes so far as to observe "that by far the *major part* of the science and art of medicine is altogether unconnected with anatomical information; and *that the actual observation of the phenomena of disease, and the operation of remedies,* is the great foundation of the physician's power and usefulness." Upon the subject of morbid anatomy, also, which he conceives has been elevated to too important a rank in our professional acquirements, he remarks that—

"Until we can discover the actions of living matter in a dead body, we cannot hope to discover the quantum of arterial influence which resides, or has resided, in an organ. It appears, then, that the action of an organ is more particularly and more immediately dependent on a principle, the existence of which we acknowledge, the agency of which we infer from its effects, but the characters of which we are wholly unacquainted with. Where is the connexion between the actions of a living and the structure of a dead body, if the anatomist is wholly unacquainted with, and has no means of recognising, that principle upon which living action is more particularly and more immediately dependent? Where is the association of the symptoms and of the disease, as discovered by morbid anatomy, if it have no possible means of recognising that principle by which the symptoms are regulated and controlled. 'It is useless to write on symptoms except as signs of diseases, and morbid anatomy is equally useless, except it be ascertainable in the living patient by such signs. In both departments of medical science, it is the accurate association of the symptoms and of the disease which constitutes at once the great difficulty and the practical utility of our investigations.'" (P. 10.)

We conceive there is much truth in these observations. It is incumbent upon every physician to be thoroughly acquainted with anatomy, and also to take every opportunity of ascertaining the *effects* of disease by an accurate inspection of morbid parts; but we would certainly deprecate an opinion that is very frequently entertained: we would by no means infer that a skilful anatomist, or one who had devoted a large share of his attention to the prosecution of morbid anatomy, must necessarily be a good physician. These are very important and very necessary qualifications, but yet we think, with Dr. Shute, that for the physician they are *minor* ones. Morbid anatomy, it must be remembered, can never discover diseases of *function,* and is therefore unconnected with those diseases which it is most essential for us to be acquainted with; "and immediately that morbid anatomy disavows all acquaintance with functional disease, it is *bonâ fide* separated from the consideration of morbid causation, because diseased function is admitted to be the primary cause of diseased organisation."

In the preliminary observations, Dr. Shute thus announces the division he adopts:

> " A pathological examination of the nervous, vascular, absorbent, muscular, and mental systems, as applicable to disease in general, will constitute the first division of my subject. Each subdivision will be preceded by an exposition of the leading pathological principles which I have espoused, and by an attempt to deduce these principles from physiological facts. The second division will comprise the application of these general principles to individual diseases, and embrace the subject of nosology." (P. 35.)

Dr. Shute considers it will not be difficult to point out some of the erroneous pathological principles which have been founded upon the assumed identity of life and living action.

> " Life and living action are distinct. If the phenomena which imply the continued operation of the vital principle, and those which indicate the existence of living action, be not always conjoined;—if "the Rotifer, which lives in small puddles of water, often on the tops of houses, shrivels up as the water evaporates, till it becomes like a piece of dried parchment, and, on being moistened with water, resume its pristine form, and become as lively as ever;'—if (in the words of Mr. Ellis,) ' these facts sufficiently demonstrate the necessity of water to the commencement of living action, and at the same time shew that life is compatible with an organisation ' shrivelled up like a piece of dried parchment;''—if, lastly, the most effectual mode of preserving life, under certain circumstances, be that of excluding the atmospherical principle, which all admit to be essential to the continuance of living action, is it not a rational conclusion that life and living action are distinct? If moisture, oxygen, and heat, be necessarily component parts of that organisation which is endowed with irritability, or susceptibility of action, and be not necessary to that organisation which is endowed with the property of life, are we not justified in saying that life is consistent with an organisation which is too imperfect for living action?" (P. 38.)

Life, according to HALLER, "est summa earum actionum *quas* singulatim describit physiologia;" according to Dr. BROWN, "is an assemblage of actions or effects;" and, in the opinion of BICHAT, is the *tout ensemble* of living actions. Death, therefore, must be a simultaneous cessation of *all*

action. "Is it not singular (properly demands Dr. Shute,) that, with such a definition of life, Bichat should have devoted his attention and labour to such enquiries as the following? How does the death of the heart produce that of the lungs? How does the death of the lungs produce that of the brain? How does the death of one organ produce that of another?" Dr. Darwin boldly asserts that "the sensorial power, or nervous energy, is life;" and yet a paralytic muscle, when deprived of the nervous influence, retains the acknowledged property of life.

Dr. Shute conceives that, if the distinction between life and living action had been preserved, the most unimportant and most questionable part of Mr. Hunter's physiology had been omitted; the luxuriant imagination of Dr. Darwin had been restrained; the hypothesis of Dr. Brown had been promulgated in a less questionable shape; and the approved theory of the present day, as it regards living action, would have been almost immediately grafted upon the discoveries of Priestley and Mayow. Dr. Fothergill was led to consider oxygen as the cause of irritability; and now, after a lapse of more than fifty years, we are told that irritability "can alone be maintained by duly oxygenised blood." This is certainly a strong argument in favour of the opinion that oxygen, modified by the powers of the animal frame, is the great and controlling agent in living action; a doctrine which is elaborately maintained in the volume before us.

"Oxygen cannot be immediately necessary to life, because the most effectual mode of preserving life, under certain circumstances, is that of excluding the atmospherical principle; but oxygen and living action are inseparable. Living action will, it is true, continue for a certain time in an atmosphere of hydrogen gas, but is supported by the oxygen previously accumulated in the system, as proved by the production of carbonic acid gas (of which oxygen is a component part,) under these circumstances." (P. 43.)

The acknowledged property of life is that of preserving the organisation of matter, both animal and vegetable, under circumstances which, but for the agency of this principle, would lead to its chemical decomposition.

"Unacquainted as we are with the nature and essence of this principle, the knowledge of its effect appears, in a practical point of view, to be 'stale and unprofitable;' if, however, a certain state of organisation be essential to the existence of this property, and if we have the means of ascertaining, and in some degree of controlling, the circumstances upon which this organisation is dependent, the vital principle, though absolutely unknown to us, is brought within the scope of practical utility; and the power of regulating the actions, upon which the organisation is dependent, is, in point of fact, a power extended to the living principle itself. If we can advance one step further, and ascertain that life is compatible with an organisation which is too imperfect for living action, a pathological fact, very extensive in its application to the phenomena of disease, and of undeniable importance in all cases of apparent death (or what has been very unphilosophically denominated suspended *animation*,) is established." (P. 43.)

That many of our readers are fully impressed with the truth of these observations, and would consequently take advantage of their knowledge in the execution of their practical duties, we doubt not; but still we apprehend Dr. Shute has done well to repeat, and to impress the importance of, a principle which is not unfrequently overlooked, and by some perhaps is altogether unknown.

There is reason to suppose that many structures of the body are altogether unendowed with susceptibility of action, presenting us with examples of life without living action, and contributing by a *mechanical* function to the support of that organisation upon which life is dependent. According to our present views, the animal frame is considered to be a compound of animate and inanimate matter, a heterogeneous mixture of dead and living substances, some endowed with extreme sensibility, and others not only reduced below the scale of living action, but unendowed with the property of life.

"This opinion is founded upon the assumed identity of life and living action, many structures of the body being evidently unendowed with susceptibility of action; and yet these structures are not obedient to the laws of chemical agency. If the distinction between life and living action be preserved, every part of the body amalgamates with each other; the incongruous union of living and dead matter is no longer assumed; and the inconsistency of supposing that animal matter can resist the agency of chemical influences, without that property to which this peculiar power belongs, is avoided." (P. 46.)

In the present day we apprehend there are very few, if any, practitioners who are so imperfectly acquainted with the laws of the animal economy, as to suppose that any part of the animal structure is so destitute of the property of life, although the degree of vitality is known to exist in various proportions in various parts. Upon many physiological points the doctrines of Mr. Hunter, much as we are indebted to his talents and improvements, are now known to be erroneous.

Dr. Shute proceeds at some length to show the necessity for, and the effects produced by, oxygen in the living body. The correspondence in many respects between animal and vegetable organisation is also pointed out. After which, having briefly touched upon the opinions entertained by different physiologists upon the subject of animal heat, our author observes—

"Consistently with these general remarks, may it not be assumed as a physiological fact, that the degree of animal heat is, in the human subject, proportioned to the quantity of the aerial principle incorporated with the system through the medium of nerves? If so, we arrive at the important pathological conclusion that the degree of heat, whether morbidly increased or diminished, whether general or local, is indicative of a corresponding nervous action." (P. 114.)

In the opinion of some writers, fever is the *effect* of arterial excitement, whilst others as warmly contend that the arterial excitement is the effect of that state of the system which constitutes fever. "I can have no hesitation (says the author,) in saying that I adopt the latter opinion, and for the following reasons:

"The remote causes of fever, acting primarily on the nervous system, may, I think, by a regular chain of evidence, be traced into arterial excitement; and such arterial excitement (as I shall endeavour to show in a future part of these observations) is the only mode by which the morbid state of the nervous system, constituting the proximate cause of fever, can be removed. I cannot, on the other hand, discover any connexion between the remote causes of fever and arterial excitement, except through the medium of the nervous system; no such connexion has hitherto been established, or successfully attempted: and upon this point the vascular pathology is, I think, altogether defective." (P. 119.)

In this view of the primary formation of fever, Dr. Shute has our support. It follows as a necessary consequence, from his having adopted this opinion, that the author is opposed to many of the doctrines of Parry, whose work upon the Elements of Pathology and Therapeutics he comments upon at some length. He confines himself to the consideration of this work, as it is the only systematic book upon the subject, and as it may be considered the text-book of the vascular pathologists. A formal refutation of the opinions of Dr. Parry might, perhaps, have been spared. There has, we believe, been but one opinion respecting the endeavours to prove that derangement of the nervous system is not as frequently the original cause of disease as derangement of the vascular system. Nothing like a satisfactory body of evidence has been brought forward in confirmation of such a doctrine, and much less is it generally conceded that we are always to look for the first movements of disease in some irregular action of the blood-vessels, independent of the nerves. ' More than enough has been said to prove "that the vascular pathology is not founded on facts 'cognisable by the senses, or arranged in their natural order;' that it is not less 'visionary or inapplicable' than the doctrine to which it is opposed; that it is not 'consonant to that simplicity which pervades all the other known operations of nature;' and that, if it be estimated by the criterion that the 'great and ultimate end of pathology is its application to practice:' it is, in no respect, more eligible, convincing, or satisfactory, than the nervous pathology." (P. 151.)

Diseased action, in fact, may belong either to the nervous, vascular, absorbent, or muscular structures.

*Febrile diseases.*—If it be admitted that the cause of power is accumulated in the brain, we are justified in stating that the accumulating function of that organ is the provision of nature, by which the capacities of the system are proportioned to its necessities, by which the equilibrium between the expenditure and the supply of power is maintained.

"If, however, the expenditure so far exceed the supply as to reduce the nervous influence below the healthy standard of accumulation,—below that standard which is capable of being restored by the ordinary actions of the system, by the periodical return of sleep and repose,—such expenditure constitutes disease, and (as we shall endeavour to show) that peculiar disease which is denominated fever. 'Quod verò plus impendit, et delassat, Labor est." (P. 201.)

That in some cases fever may be thus produced, we grant; but we do not think the author has succeeded in establishing it as a general principle. The nervous influence is frequently reduced "below the healthy standard of accumulation," but yet no fever results. It may be reduced "below that standard which is capable of being restored by the ordinary action of the system, or by the periodical return of sleep and repose," and disease may arise; but the disease thus established frequently does not exhibit that aggregation of symptoms to which, with any accuracy or propriety, the term fever can be applied. The sufferer may become debilitated in mind and body; he may exhibit all the usual symptoms of mental and corporeal exhaustion; but he may not labour under symptoms to which any species of fever can lay claim. We cannot, however, enter into a formal consideration of the various arguments which Dr. Shute brings forward, and ingeniously maintains, in support of an opinion which we allow may sometimes be correct, but which, we contend, will be found frequently defective and erroneous.

Dr. Shute appears to be aware that some difficulty exists to show that fever arises invariably from exhaustion, and he thus argues the question:—"In the exhaustion of fatigue, the diminished atmospherical expenditure of repose is often of itself sufficient to restore the brain to its natural standard of accumulation, and then no febrile paroxysm, supported by an inordinate determination of the nervous influence to the capillary extremities, will occur; or the object will be so readily accomplished, that the febrile reaction will be scarcely perceptible. If, however, the exhaustion has been excessive, a febrile paroxysm, continuing until the energy of the brain is restored, will be perceptible and evident." That under such circumstances a febrile reaction may occur, we readily admit; but it will not follow as a necessary consequence of previous exhaustion, however excessive it may have been. The author indeed himself, in a subsequent page, observes, "that all kinds of debility are not productive of febrile reaction is a fact too evident to be disputed."

Upon the subject of the connexion of inflammation with fever, Dr. Shute thus states his opinion, with which we concur.

"It is an acknowledged fact that fevers not unfrequently occur, and run their progress, without any morbid disorganisation; and it is equally true that such disorganisation not unfrequently takes place. The fever, whether accompanied with morbid disorganisation or not, is always regulated by the state of the brain, or the powers of the system to answer its necessities. Whether the inflammation of a particular organ occur previously or subsequently to the accession of a febrile paroxysm, the fever is immediately dependent on the state of the brain, and the inflammation is to be regarded as a remote cause, or an effect, not constituting in either instance an essential part of fever. 'Does inflammation never take place in fever? Most certainly it does, and that very frequently; but the inflammation, whether of the brain, of the membranes, or viscera, is not primary and characteristic, but secondary and incidental. The identifying, then, of fever and inflammation is an instance of modern generalisation, which does not appear to me to be countenanced by fact.'" (P. 343.)

*Paralytic diseases.*—There are two circumstances or conditions necessary to every vital action,—viz. the impression of an agent, or stimulus by which the action is excited, and the power of being excited into action by the stimulus applied. The latter power or property has been denominated irritability; and it is on some change in the irritability of the organs affected that those diseases are depending, to which Dr. Shute next invites the attention of his reader.

A state of permanent tension in the fibres of the body is necessary for the existence of life, and any undue departure from such condition is followed by debility.

"Relaxation and debility of the living fibre may, according to this view of the subject, be regarded as synonimous terms; and hence the diseases of this class have been denominated paralytic, the word being used in its literal and extended sense, and meant to include all those general affections of the system which are characterised by a diminution of power arising from diminished irritability of the organs affected. Fever, it may be remarked, is also characterised by diminution of power, and such diminished power is referable, according to the principles advanced in the last chapter, to diminished irritability. But fever is a compound of increased and of diminished actions; and the former having been universally selected as the leading features upon which to establish the classification of febrile diseases, we neither saw the necessity or advantage of deviating from the ordinary course. There is, however, in point of fact, a much nearer connexion between febrile and paralytic affections than has generally been imagined; but, as the increased actions are more developed in the former, and the diminished actions in the latter, this circumstance has been selected as the basis of their nosological arrangement; and, therefore, by the term paralytic diseases we mean to designate all those general affections of the system which are characterised by a diminution of power arising from diminished irritability of the organs affected, and are at the same time unaccompanied by fever." (P. 347.)

The classification of CULLEN is objected to by Dr. Shute, who divides the *neuroses* into two classes,—into those of diminished and of increased action.

"Fever, as we have endeavoured to show, is an example of increased action connected with morbid exhaustion, and spasm is an example of increased action arising from inordinate accumulation of the atmospherical principle. Paralysis, on the contrary, is a diminished action arising from a morbid deficiency of the aerial principle, and, generally speaking, from an exhausted brain: if, therefore, we look to the proximate cause of febrile and of paralytic affections, we find it to be the same; and, if we extend our observations to the symptoms, we cannot fail to remark that diminished power is a characteristic feature of both classes of diseased actions. These circumstances are, indeed, so modified as to justify us in referring febrile and paralytic affections to different classes; but a modification of circumstances implies a degree of similarity, which enables us to pass from the consideration of the former to that of the latter diseases, without losing sight of those general principles which are found to regulate the actions of the system in all cases of cerebral exhaustion." (P. 350.)

It is obviously necessary to have a clear comprehension of the term irritability, before we can satisfactorily proceed to the consideration of a class of diseases in whose description it must be so frequently employed. The power of this term is an old bone of contention, and we beg to refer to some observations in a recent Number* on the various acceptations which have been attached to it by different authorities.

Dr. Shute thus defines it:—"It is that property of living organised matter by which it becomes susceptible of action; that it is dependent on the atmosphere; that it is intimately connected with the aerial principle, derived through the medium of arteries and nerves; that it is a component part or property of the organ whose action it supports, and is expended by the action itself." (P. 357.)

"It may be said, according to the author, in general terms, that an interruption to the supply of oxygen is an interruption to the supply of irritability, and that in asphyxia every function, both mental and corporeal, is paralysed, because that upon which every function is more or less immediately dependent is interrupted." In a pathological point of view, however, it is considered necessary "to ascertain and account for the changes which are going on in the system preparatory to the general paralysis, not only because those changes are the more immediate causes of the suspended action, but because a knowledge of these changes will be found more or less applicable to the various modifications of paralytic diseases."

Paralytic diseases have been improperly confined to the brain alone, because the effect is precisely the same whether the cause of irritability be interrupted in the lungs, in the circulation, or in the brain.

"Asphyxia is an example of paralysis originating in the lungs' but the various symptoms of this complaint are immediately referable to an interruption of the atmospherical principle in the lungs, the heart, or the brain. Syncope, though in many respects a nervous disease, is in its leading features referable to the class of paralytic diseases. Diminished energy of the brain is the immediate cause of apoplexy. Asphyxia, syncope, and apoplexia may all be traced to modifications of the same proximate cause. If febrile and paralytic affections are referable to the same cause—to morbid deficiency of the atmospherical principle, it is natural to inquire if they at all correspond in other respects. There is no

* See our analysis of MAYO's Physiology, No. for April.

vascular excitement in asphyxia, because the brain is exhausted to a point which is incompatible with the support of febrile reaction. There is no vascular excitement in syncope, because the deficiency of the atmospherical principle is partial, and does not involve that cerebral exhaustion which renders febrile reaction a necessary and salutary process, or because the brain, for want of its accustomed stimulus, is incapable of directing its energies to the support of the febrile reaction. The vascular excitement of apoplexy is imperfectly developed, because the brain, being more exhausted than in fever, is unequal to the support of the febrile reaction. The febrile process corresponds with the different gradations of cerebral exhaustion; the first degree being relieved by the suspension of the mental function alone, as in natural sleep; the second by diminished energy of the functions of waste, conjoined with vascular excitement, as in the fever of health; the third by a greater diminution of the functions of waste, and greater excitement of the functions of supply, as in the fever of disease. If the exhaustion be still greater, the functions of waste are absolutely suspended, and the vascular excitement, corresponding with the state of the brain, is imperfectly developed, as in the last stages of fever and in apoplexy. A total suspension of the functions of waste and of supply characterise that extreme degree of cerebral exhaustion, which is altogether incompatible with the support of febrile reaction, and consequently of life, as exemplified in asphyxia. The necessity of febrile reaction to an exhausted brain is thus equally exemplified in febrile and paralytic affections, such diseases being always fatal when the brain is exhausted to a point which is incompatible with the support of the febrile process." (P. 442.)

In the last chapter Dr. Shute treats of Spasmodic Diseases; but our limits compel us to draw our article to a close.

It would be unjust to dismiss the present work without complimenting Dr. Shute upon the ability and ingenuity of argument displayed in it. It is much more, however, calculated for the practitioner, who has laid in a sufficient store of fundamental knowledge to enable him to appreciate the frequent critical examination of the opinions of various authors with which it abounds, than for the student, who seeks rather for practical conclusions than theoretical disputations, however ably and satisfactorily they may be conducted.

---

*Dublin Hospital Reports and Communications in Medicine and Surgery.* Vol. IV.—8vo. 15 Plates. Hodges and M'Arthur, Dublin; Longman and Co. London, 1827.

The Dublin Hospital Reports have been very creditable to the practitioners of the sister kingdom, and have added very considerably to our stock of medical and surgical information: we are therefore glad once more to find another volume on our table, after an interval of rather alarming duration.

The editorship, we believe, has devolved upon Dr. CUSACK and Dr. COLLES, who, after alluding to the difficulties which have hitherto impeded them, announce "that several professional gentlemen, who have already contributed to the reputation of this work, have been prevailed on to join with them in undertaking a new series, the first volume of which may be expected in the course of next year." The present volume, which would thus appear to terminate the first series, contains numerous papers of considerable, and some of high interest. The subject most worthy of attention in the work is the Report of the Amputation of portions of the Lower Jaw, by Dr. CUSACK, and Cases of Excision of a portion of the Lower Jaw, by Mr. CRAMPTON. Some instances are recorded in which this operation has been performed in Germany, in America, and more recently in this country. But the papers before us contain much more extensive and satisfactory information than any which have preceded them.

As the multifarious nature of the contents renders any thing like a critical analysis of the volume unconnected, and therefore unsatisfactory, we shall give the most interesting facts which it contains in our COLLECTANEA in this and succeeding Numbers, arranged according to subjects: and we may add, that by this plan we shall be enabled, from the smallness of the type, to give much more copious extracts, and to do more justice to the many valuable papers which the volume contains.

---

*A Treatise on Clinical Medicine, being a Compendious and Systematic Introduction to Practice, as contained in the Memoranda of* I. R. BISCHOFF, M.D. Imperial Professor of Clinical Medicine, Physician to the General Hospital, and also to the Lying-in Hospital in Prague. *From the German,* by JOSEPH COPE, M.D. 18mo. pp. 280. Anderson, London, 1827.

THIS little volume contains the histories of 148 cases, recorded, we nothing doubt, with the utmost "veracity and candour," and is therefore of considerable interest, as showing the state of medical practice at the clinical school at Prague. Nevertheless, whoever expects to find it what the title-page announces, "a Treatise on Clinical Medicine," will be disappointed, as it consists of little more than notes of cases following each other without any methodical arrangement, and occasionally accompanied with a few cursory remarks.

One circumstance, however, deserves notice: we allude to the superiority of the system of clinical education on the continent as compared to that adopted in this country.

"But in order to accomplish in the most certain manner the great object of introducing the student, who has already gained the necessary theoretical information, into the career of practice, and to form him (according to that sphere of it which falls to the surgeon's lot,) for a conscientious, useful, and prudent discharge of his duty, the following plan is adopted in the clinical school:—Every patient, when admitted, is made over to a student, as his ordinary, who then publicly, and in the presence of the professor, makes, with all possible exactness, that so essential examination of the patient, by which the following are made out in the most careful manner:—1st. The patient's habit of body, and an account of his previous maladies. 2d. The exciting causes. 3d. The commencement and course of the disease to that period. 4th. The symptoms of the disease at the time of admission. 5th. The remedies already applied, and their effects." (P. xiii.)

"All that in this short review has been set down respecting the patient, is now put down by the ordinary in writing, and in the same order, with truth and clearness, and read out publicly on the following day, as the history of the disease, or pathologic biography. During the course of the disease, it becomes the duty of the ordinary daily, before the professor's visit, to examine the patient, and to set down in writing the changes that may have taken place, and the general appearance of the disease. On the opening of the clinical school, these observations are read out, their truth confirmed, their errors corrected, and the whole amended.

"By this a two-fold object is attained:—1. The student himself learns to make observations. 2. A bar is put to the making out the patient's case, after perhaps the lapse of some days, and often from an uncertain memory; by which occasion is given to such extraordinary and scarcely credible accounts of disease. In this manner the treatment is continued till its termination in a cure or death. In the last case, the post-mortem examination takes place; previous to which, in reference to the disease, the morbid appearances most likely to present themselves are stated. During the examination, these are noticed with the greatest attention, and consigned to writing. The whole is now compared with the history of the disease and the previous diagnosis; the account of the examination is again publicly read on the following day; the true or mistaken judgment exposed, and the practical results deducible from it particularised." (P. xvi.)

The deficiency of clinical instruction in London is unquestionably the greatest imperfection in the medical education of the metropolis, and the remark is more applicable to the department of physic than surgery. In London, the surgeon is followed through the wards of his hospital by a crowd of pupils, the physician by comparatively few. This is generally attributed to the more visible and tangible nature of surgical cases, which, being more obvious to the senses, require less attention, and prove more attractive to the student; and there can be no doubt that these circumstances partly occasion the difference. Yet we are convinced that it is also in a great measure dependent on the fact that the interest is kept up among surgical pupils by occasional clinical remarks, or regular lectures; and we have observed that those surgeons are most followed who adopt this latter method. When the physicians are admitting patients, there are always abundance of pupils present, because at such times they hear questions regularly and methodically put: their attention is thus directed to the nature of the case,—they have an opportunity of making distinct notes,—and they see the application of the remedies accommodated to the indications which the previous history has afforded. Let the same thing be done every day that the physician sees the patient,—let the previous report be read at the bedside, and the report of the day be detailed; and this plan, simple as it is, would add much to the interest of the pupil, even if followed by no clinical lecture. We feel satisfied also that it would increase the number of physician's pupils, and remove much of the difficulty in acquiring a competent knowledge of medical practice.

But to return to Dr. Bischoff: we fear his work, in itself rather desultory, and indifferently translated, will not tend much to excite that taste for clinical instruction which we regard as so desirable. The following case, which is in itself not devoid of interest, will show the general manner of the author, and the style of his translator:

"The following uncommon and interesting case came on in the form of an inflammation of the thyroid gland:—Anna John, twenty-four years of age, a nurse, of a middling stature, weaned her child on the 16th of December, 1822; two days after, she had head ache, heat, and cold, with complaints in the neck, for which she took some medicine at home.

On the 20th, she came into the hospital in the following state:—She had pain at the back of the head, her countenance was pale and sunk, her look melancholy, her thirst increased, her voice weak, deglutition impeded, but without traces of angina; the tongue somewhat white. On the neck externally, the thyroid gland was enlarged, the skin stretched, and painful to the least touch; on a deep inspiration, she felt a darting pain in the fore part of the breast, without any cough being excited; at the same time there was a constant inclination to vomit, though she felt no pain from pressure of the stomach; the urine was rather high-coloured; she had had two loose evacuations; the warmth of the skin was moderately increased; the pulse frequent, oppressed, and small; she felt herself, moreover, very much cast down.

"The disease was, as to its form, considered to be an inflammation of the thyroid gland, yet the troubled, pale countenance, the great debility, the contrast in the symptoms,—viz. the impeded deglutition, without any inflammation or other mechanical impediment, the almost extinguished weak voice, the pains of the chest, without the other signs of inflammation; the constant inclination to vomit, with gastric impurities or pain at the stomach; the circumstance of an oppressed small pulse, seemed to warrant the conclusion that it bore rather the character of a nervous fever. But, if one considers the tenor of all the symptoms collectively, and compares them with the physiological destination of the organs, it will appear that the pain in the occiput, the peculiar derangement of the vocal organs and of the deglutition, also of the expectoration and stomach, announce a morbid affection of the nerves supplying the neck, chest, and abdomen as the cause; and from the deranged functions of which, from inflammatory irritation, all the symptoms are satisfactorily explained. Hence, a rather antiphlogistic treatment was directed against this inflammatory irritation; six leeches were applied to the thyroid gland, and along the neck emollient fomentations; Decoct. Salep; and every three hours half a grain of Calomel was administered. In the evening, as the tense pain of the gland continued, six more leeches were applied, and mercurial ointment was rubbed in on each side of it.

"On the 21st, her state was, after a rather unquiet night, much the same, only the constant inclination to vomit had diminished. A blister was now applied to the nape of the neck. Towards the evening, the deglutition was easier; vomiting still took place, by which a little phlegm was thrown up. For this, powders with four grains of Magnesia, as much sugar, Calomel and Hyoscyamus each half a grain, were given every three hours.

On the 22d, the swallowing was still better, and the vomiting had ceased by the use of the powders; the swelling and pain of the thyroid gland were considerably less; the patient had four loose stools; the urine threw down a mucous sediment; the pulse was still frequent, yet more free, and sufficiently strong.

"Dec. 23d, all the symptoms are milder; the gland is almost free from pain, but hard; the patient has a cough. The same on the 24th, though at night a troublesome vomiting came on again, and she suffered a good deal from the cough and mucous expectoration. She now took powders with half a grain of Hyoscyamus; Ung. Hydrarg. to the region of the stomach; and a blister was applied to the sternum. On the following day she experienced much relief; though, on the 27th, the gland seemed to be more swollen, tense, and painful; the cough continued, though the breathing was perfectly free. Four leeches to the gland, and emollient applications were employed; she continued the salep decoction. In the days which followed she improved, though, on the 30th, she felt again severe pains in the gland, for which four leeches were applied. Finally, by the diligent use of emollient cataplasms, and inunction of mercurial ointment, the inflammatory disposition of the part was overcome, and it was restored to its natural size; so that, on the 4th of January, the patient was quite well.

"On that day, however, towards evening, without any cause, she had a smart attack of fever, with shivering and heat, which returned on the following day at the same hour. We looked for an intermittent fever, and merely observed its progress, but she had no return of it; and on the 15th of January she left the hospital cured.

---

